# Editorial: New information from and about the KiGGS study

**DOI:** 10.17886/RKI-GBE-2018-020

**Published:** 2018-03-15

**Authors:** Bärbel-Maria Kurth

**Affiliations:** Robert Koch Institute, Berlin, Department of Epidemiology and Health Monitoring

This issue of the Journal of Health Monitoring focuses on one main topic: presenting the very first results from KiGGS Wave 2. After the first population-based health examination survey for children and adolescents (KiGGS baseline study) and its first follow-up survey (KiGGS Wave 1), many people are looking forward to the results of KiGGS Wave 2 – not just epidemiologists, but also (health) policy makers, paediatricians, representatives of the public health service, health insurers and also many parents. As the people responsible for planning and implementing KiGGS Wave 2, we certainly feel the same way. The eagerness with which everyone is awaiting the results reflects the relevance of this study. Information about the effectiveness of interventions and preventive measures, about whether the goals and sub-goals defined in the national health target ‘Growing up healthy’ have been achieved, and about developments in health during the transition from adolescence to adulthood can help deliver a strong foundation for the future promotion of the health of children and adolescents living in Germany. We could have continued working on the preparation and quality assurance of our data for several more months before presenting as many findings as possible as part of a single ‘major’ project. Instead, we have decided to hold a public symposium under the motto ‘New information from and about the KiGGS study’ on March 15, 2018 and to publish the first findings from KiGGS Wave 2 in this issue of the Journal of Health Monitoring. It will be followed up by a series of further issues that set out the results.

The articles in the current issue on the ‘cross-sectional’ and ‘longitudinal’ components of KiGGS (KiGGS Wave 2 cross-sectional study – participant acquisition, response rates and representativeness, KiGGS Wave 2 longitudinal component – data collection design and developments in the numbers of participants in the KiGGS cohort) lay the foundations for understanding and classifying further results from KiGGS Wave 2. This is essential because KiGGS is a highly complex study consisting of two components and two different concerns.

On the one hand, we want to present representative findings about the health and health-related behaviour of the next generation: how high is the proportion of overweight or obese children and adolescents? Has this percentage continued to increase over the last eleven years or has the increase been stopped? Do more or fewer teenagers smoke today? Which developments have taken place in relation to physical activity? In short: is the health of children and adolescents in Germany getting better or worse? And, have prevention measures already begun to show results? Our population-based cross-sectional data can be used to draw conclusions about trends such as these. As part of the health monitoring framework, KiGGS is focused on one of the most important fields of action in public health: monitoring the health and the health-related behaviour of a vital section of the population – children and adolescents. Studies such as KiGGS always leave us better informed than we were before they were undertaken; for instance, changes in the educational and social structures always have an impact on health and they are reflected in our results.

The other – no less important – set of issues covered by the study are the developments in health that occur during the life course. If we are to understand when is the most favourable window to implement preventive measures and interventions or to set the course for health-related behaviour in years to come, we will need more than just a description of trends. What are the chances of an obese child having a normal weight during adulthood? At what age is smoking particularly dangerous, because after that, it becomes very difficult to quit? Is there a specific influencing factor, which, in the presence of allergic sensitisation, later leads to allergic diseases and the symptoms that they are associated with? These questions can be answered using data from the second component of KiGGS Wave 2 – the KiGGS cohort.

Data for ‘our’ KiGGS cohort is collected from the children and adolescents who took part in the KiGGS baseline study between 2003 and 2006. During the baseline study, we asked the participants whether they would be willing to take part in the cohort – we received an overwhelmingly positive response. Nevertheless, it was and will continue to be difficult to integrate people who are now between 10 and 31 years of age into our study. This problem was further complicated by the study design of KiGGS Wave 2: in order to improve field-work efficiency and minimise costs, the examinations were conducted in the same 167 sample points used for the baseline study. There, examinations were undertaken for the participants of the new cross-sectional sample as well as the children, adolescents and young adults from the KiGGS cohort. This decision came with a price: on the one hand, it made the work of the field teams, the study office and the home visits prior to the survey enormously complex, employing various instruments during the examinations and interviews required a lot of concentration and could not be allowed to have an impact on each of the other parts of the study. It was not possible to achieve this equally well in every case and at all locations. On the other hand, establishing examination centres in the 167 sites where the KiGGS baseline study was conducted came with a disadvantage for the young adults belonging to the cohort, as they were often unable to participate in on-site examinations. Young adults are a highly mobile group and a large proportion of the cohort no longer lives in the locations where they were examined for the baseline study – the 167 places of residence at the beginning of the study have now transformed into almost 2,000. As such, we could only write to the participants who had moved away, send them a questionnaire, and ask them to return it. Unfortunately, in many cases this meant that a central aspect of the study was missing – the examination. A lot of effort was put into encouraging the participants to fill out the questionnaire online within an extension period. Since the cohort is such a valuable component of KiGGS and remains unique in Germany, the follow-ups were only finished in August 2017. This is why for the KiGGS symposium and this issue of the Journal of Health Monitoring, analyses on topics from the interviews could only be performed using data from the KiGGS baseline study and KiGGS Wave 1. The cohort interview data from KiGGS Wave 2 are simply not ready. For the participants who were examined in the study centres, however, the cohort data set from KiGGS Wave 2 was ready earlier, and this means that it was possible to calculate transition probabilities for overweight and obesity, for example. For the participants who also submitted a blood sample, laboratory parameters could be analysed to study developments that had occurred between the two points in time. The corresponding ‘laboratory data set’ has got again a different sample size. In order to spare Journal readers and participants at the KiGGS Symposium any confusion, the various data sets – including sample sizes – are set out in Table 1.

In addition, the urgent need to be able to plan the future of the KiGGS study also influenced our decision to publish results from both the cohort and the cross-sectional component in a timely manner. We will be able to run KiGGS as a cross-sectional study and finance it with the help of the Federal Ministry of Health as long as observing the health of children and adolescents in Germany continues to be a defined task within the framework of Federal Health Monitoring. At the same time, the participants of the KiGGS baseline study will probably all be of adult age by the time the next wave is conducted; strictly speaking, this will make the study’s subtitle ‘German Health Interview and Examination Survey for Children and Adolescents’ obsolete. It is clear that this part of KiGGS can no longer be funded using financing from our health monitoring system. However, in our view, and from the perspective of public health, this part of the study is very important, highly valuable, and unique even at the international level. We will also have to change the design of this part of the study (which brings to an end the principle of undertaking examinations in examination centres) and employ different methods to those that have been used so far (will we succeed in decentralising measurements, obtaining information using health apps and binding young adults to the study through cleverly chosen incentives?). As such, the questions employed in the study and the methods of analysis will change; in fact, this has already begun to take place. The first three waves of examinations and interviews, which also contain information obtained through the parents, generated about 200 million data points. We are currently in the process of using a doctoral project to develop new methods of digital epidemiology, machine learning and pattern recognition to identify correlations at the descriptive level. We then intend to model and test the results using classical epidemiological methods. Incidentally, our cohort dataset holds so much potential and is so complex and valuable that we will soon begin to undertake joint analyses through cooperation agreements as part of in-depth analysis projects.

In addition to information on health status and health-related behaviour, which we would very much like to continue collecting for life course research, we will also be adding new content that reflects the changing living and working environments of young adults. We are highly enthusiastic about our work and have numerous new ideas that we would like to implement. The sword of Damocles hanging over the study – our lack of funding – means that we will have to consider all of the options for financing if we are to continue the cohort study.

In the meantime, and until we solve the problem of financing, we will continue to do our utmost to hold on to our ‘original KiGGS participants’, to contact them and to interest and motivate them with our exciting results. We have also produced a results booklet from the findings published in this issue, and we would be very happy to send you a copy. Finally, if you have any further ideas about how KiGGS could be continued, we would very much like to hear about them.

Bärbel-Maria Kurth

Head of the Department of Epidemiology and Health Monitoring at the Robert Koch Institute

## Figures and Tables

**Table 1:** Overview of the numbers of participants in the KiGGS cross-sectional study and the KiGGS cohort according to study wave Source: KiGGS baseline study (2003-2006), KiGGS Wave 1 (2009-2012), KiGGS Wave 2 (2014-2017)

	KiGGS baseline study (cross-sectional and longitudinal components)	KiGGS Wave 1 (cross-sectional component)	KiGGS Wave 1 (longitudinal component)	KiGGS Wave 2 (cross-sectional component)	KiGGS Wave 2 (longitudinal component)
**Age group (years)**	0-17	0-17	6-24	0-17	10-31
**Participants in the study (total)**	17,641	12,368	11,992	15,023	10,853
**Participants in interviews and examinations**	17,641	–	–	3,567	6,465
**Participants with blood tests**	14,386	–	–	3,016	6,044
**Cohort participants with an interview**	100%	–	68.0%	–	61.5%
**Cohort participants with an interview and examination**	100%	–	–	–	36.6%

